# Spatial inequalities leave micropolitan areas and Indigenous populations underserved by informal STEM learning institutions

**DOI:** 10.1126/sciadv.abb3819

**Published:** 2020-10-09

**Authors:** Rachel A. Short, Rhonda Struminger, Jill Zarestky, James Pippin, Minna Wong, Lauren Vilen, A. Michelle Lawing

**Affiliations:** 1 Department of Ecosystem Science and Management, Texas A&M University, College Station, TX, USA.; 2 School of Education, Colorado State University, Fort Collins, CO, USA.; 3 Department of Wildlife and Fisheries Sciences, Texas A&M University, College Station, TX, USA.

## Abstract

Informal learning institutions (ILIs) create opportunities to increase public understanding of science and promote increased inclusion of groups underrepresented in Science, Technology, Engineering, and Math (STEM) careers but are not equally distributed across the United States. We explore geographic gaps in the ILI landscape and identify three groups of underserved counties based on the interaction between population density and poverty percentage. Among ILIs, National Park Service lands, biological field stations, and marine laboratories occur in areas with the fewest sites for informal learning opportunities and have the greatest potential to reach underserved populations, particularly in rural or high poverty counties. Most counties that are underserved by ILIs occur in the Great Plains, the southeast, and the northwest. Furthermore, these counties have higher Indigenous populations who are underrepresented in STEM careers. These unexpected geographic gaps represent opportunities for investments in ILI offerings through collaborations and expansion of existing resources.

## Teaser

Investments in informal STEM should target underserved counties to reach populations underrepresented in STEM careers.

## INTRODUCTION

Public understanding of science requires more than content knowledge; it also requires knowledge of the nature of science and positive beliefs about science (*1*). A comprehensive understanding of science supports a better informed public that can make evidence-based decisions (*2*) and contributes to a healthier population; greater interest in Science, Technology, Engineering, and Mathematics (STEM) careers; and higher earnings (*3*–*5*). Unfortunately, access to information is unequal, with rural and poor communities receiving the fewest programs for public education in science and science literacy (*6*–*8*). Consensus from the National Academies of Sciences, Engineering, and Medicine (*5*) refers to science literacy as “familiarity with the enterprise and practice of science” [also see (*9*)]. Investments in improving public science literacy have historically focused on the classroom (*10*); yet, with most people in the United States spending only an estimated 5% of their lives inside of formal classroom settings (*11*), informal learning experiences can be valuable for science literacy, especially for adults (*12*, *13*).

In contrast to a classroom’s formality, places of informal learning provide opportunities for visitors to learn through inquiry (*14*) and educate the public through engaging experiences (*12*). Informal learning is recognized as “including learner choice, low consequence assessment, and structures that build on the learners’ motivations, culture, and competence” (*4*). Informal learning occurs in homes, at work, through digital media, and within dedicated institutions and outdoor spaces. Inclusion of a variety of learning environments that complement each other produces a richer STEM learning ecosystem (*15*). As such, informal learning institutions (ILIs) are important places where informal STEM learning occurs (*4*). ILIs, such as museums and science centers, increase appreciation for science (*16*), increase understanding of the nature of science (*14*), and positively influence attitudes and beliefs about science and technology (*12*). Other STEM-related ILIs include botanical gardens and arboretums, zoos and aquariums, public libraries, National Park Service (NPS) lands, and biological field stations and marine laboratories (FSMLs). Although visitor experiences differ across types, ILIs comprise a geographic landscape of informal learning opportunities for the general public.

ILIs can make STEM knowledge relevant, accessible, and meaningful (*15*), which can be especially important for members of underrepresented groups in the sciences who may feel excluded from informal STEM learning (*17*, *18*) or who may not recognize the viability of STEM careers (*5*, *19*). Minorities, girls and women, and rural and poor populations are persistently underrepresented in the sciences (*8*, *20*, *21*). Creating informal STEM education opportunities within underserved areas and for underrepresented groups can reduce barriers, promote science literacy, and contribute to better representation in STEM careers (*4*). While close proximity removes a distance-based obstacle, it does not ensure equity or even accessibility. Many people visit ILIs annually (*22*) and are willing to travel to do so (*23*), but structural barriers, such as entry and day trip costs, and socially exclusive practices related to class and ethnicity limit the diversity of ILI audiences (*17*, *18*, *24*). As a consequence, and despite these obstacles, to realize the benefits of broadening participation in STEM via ILIs, we first need to know the populations that currently are least able to participate in informal STEM learning opportunities because of distance.

Here, we map ILIs in the United States and explore their relative densities in the informal learning landscape to determine the national geographic distribution of each type. We identify ILI deserts where there are currently fewer sites for informal STEM education and more opportunities for ILI development, collaboration, and expansion of existing resources. We expect counties with higher population densities to have more ILIs because of larger potential audiences and counties with higher poverty to have fewer ILIs because of fewer financial resources. In addition, we explore the racial and ethnic demographics of underserved counties to determine whether populations with few informal learning opportunities are also those populations who are underrepresented in STEM careers.

## RESULTS

**Fig. 1:**
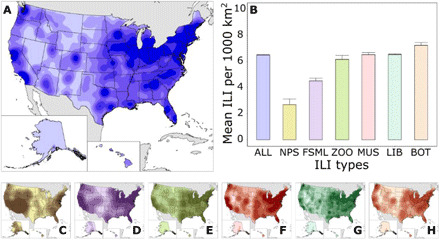
Landscape of ILIs in the United States (density per 1000 km^2^). (**A**) Kernel density surface of all ILIs displayed as six quantiles (cell size = 1000 m^2^; μ = 6.5 ILI per 1000 km^2^). (**B**) Mean density of ILIs per 1000 km^2^ at each type (abbreviations follow) with SE bars [colors correspond to (A) and (C) to (H)]. (**C**) National Park Service (NPS) lands. (**D**) Biological field stations and marine laboratories (FSMLs). (**E**) Zoos, aquariums, and wildlife conservation (ZOO). (**F**) Science museums, children’s museums, and planetariums (MUS). (**G**) Libraries (LIB). (**H**) Botanical gardens, arboretums, and nature centers (BOT). All densities are displayed as six quantiles, and values associated with each break are in table S4. Mean values for the bar plot are provided in table S5. All ILI point data with associated kernel density values are in data file S1.

Our results document the landscape of ILIs across the United States and indicate geographic gaps across the Great Plains, the southeast, and the northwest (Fig. 1A). NPS lands and FSMLs occur in areas with lower ILI densities than other ILI types; botanical gardens occur in areas with the highest ILI densities (*F*_5,20278_ = 22.37, *P* < 0.001; Fig. 1B). Geographic distributions vary depending on the type of ILI (Fig. 1, C to H); NPS lands (Fig. 1C) are much denser in western states and along the East Coast, whereas botanical gardens (Fig. 1H) are sparse across the interior west and dense on the coasts. The density of libraries most closely reflects the overall density of all ILIs.

**Fig. 2:**
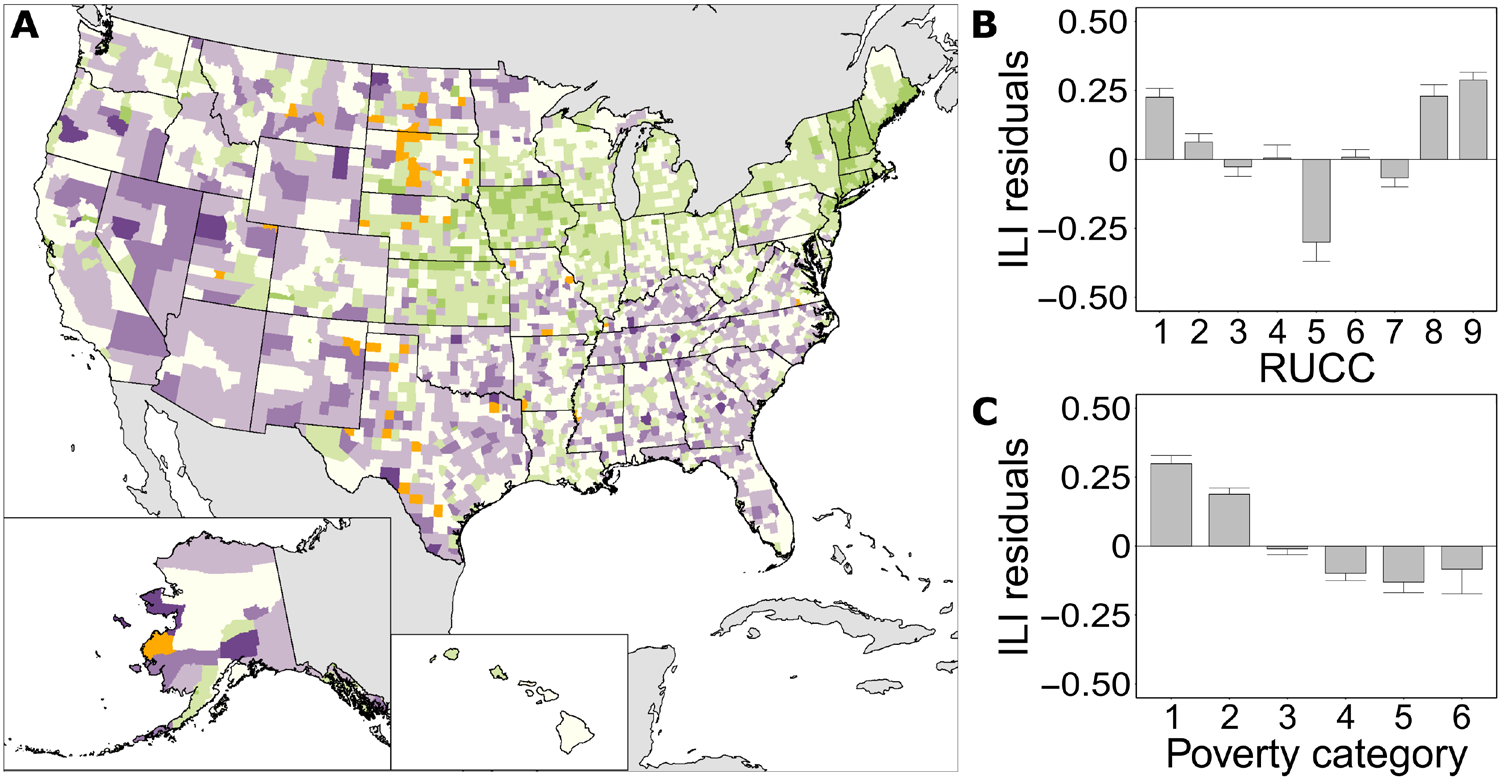
Distribution of ILIs among the U.S. population summarized at the county level. (**A**) SD of ILI residuals with the darkest purple indicating the fewest number of ILIs (σ < −2.5) and the darkest green indicating the most ILIs (σ > 1.5) relative to the number expected. Orange counties have no ILIs. (**B**) ILI residuals grouped by RUCCs with SE bars. (**C**) ILI residuals grouped by poverty categories with SE bars. ILI residuals are from a spatially corrected regression between log ILI density and the interaction of log population density and poverty percentage. Residual values associated with each SD unit are in table S6, and county data are in data file S2.

There are 48 counties with no ILIs, and these counties are primarily in the middle part of the country from North Dakota to Texas (Fig. 2A), which leaves 327,121 people underserved (0.10% of the U.S. population). Low densities of ILIs are in the southeast and the Intermountain West (Fig. 1A), and high densities of ILIs are in the Northeast, near the Great Lakes in the Midwest, and along the West Coast. These geographic patterns are related to population density and poverty levels in those areas. When considered together, population density, poverty, and their interaction explain ILI density (*R*^2^_McFadden_ = 0.322, *P* < 0.05; Table 1). Counties with low population density and high poverty have fewer ILIs. Residuals of a general linear model with a rational quadratic correlation structure to account for spatial autocorrelation are higher in the northeast and across the central Midwest, and residuals are lower in the southeast and the Intermountain West (Fig. 2A). There are 21 counties in the lowest 0.5% of ILI residuals (σ < −2.5), and these counties are primarily in the southeast and northwest (Fig. 2A). These counties have a total of only 29 ILIs (μ = 1.4 ILIs per county) and include 1,372,650 people or 0.43% of the U.S. population.

**Table 1:** Summary of the generalized linear model that included log population density, poverty percentage, and their interaction as factors with a rational quadratic correlation structure to account for spatial autocorrelation (*R*^2^_McFadden_ = 0.322).

**Factor**	**Coefficient**	***t* value**	***P* value**
Intercept	−7.63	−74.9	<0.01
Log population density	0.468	26.9	<0.01
Poverty percentage	−0.014	−4.35	<0.01
Log population density × poverty percentage	0.006	6.46	<0.01

ILI density residuals were further used to investigate which counties are more underserved by ILIs than expected based on Rural-Urban Continuum Codes (RUCCs) and poverty percentages. RUCC 1 includes counties in the largest metro areas (more than 1 million people) and has large positive residual values, which indicates that these counties have more ILIs than expected for their population densities and poverty levels (Fig. 2B). Other metro counties with 250,000 to 1,000,000 residents (RUCC 2) and fewer than 250,000 (RUCC 3) have nearly as many ILIs as expected. Counties that are completely rural or have an urban area with less than 2500 people have more ILIs than expected, regardless of whether they are adjacent (RUCC 8) or not adjacent to a metro area (RUCC 9). Most non-metro counties have nearly as many ILIs as expected. This occurs in counties with an urban area of 20,000 people or more that are adjacent to a metro area (RUCC 4) and in counties with an urban population of 2500 to 19,999 that are either adjacent (RUCC 6) or not adjacent (RUCC 7) to a metro area. The exception is RUCC 5, which includes counties with an urban area of 20,000 people or more that are not adjacent to a metro area. RUCC 5 counties have the largest negative ILI residuals (μ = −0.30, σ = 0.67) and, therefore, the fewest ILIs relative to the expectation based on their population densities and poverty levels. The 92 RUCC 5 counties include 5,028,805 people (1.6% of the U.S. population).

There is an inverse relationship between ILI residuals and poverty, so that counties with a low poverty percentage (poverty category 1) have large positive residuals and more ILIs, whereas counties with a high poverty percentage (poverty category 6) have large negative residuals and fewer ILIs (Fig. 2B). Poverty category 5 (23.6 to 31.4% poverty) has the lowest residuals and fewest ILIs; this group includes 1,229,241 people (0.38% of the U.S. population). However, none of the poverty categories have a mean residual value as low as RUCC 5 counties (Fig. 2, A and B).

### Underserved counties

**Fig. 3:**
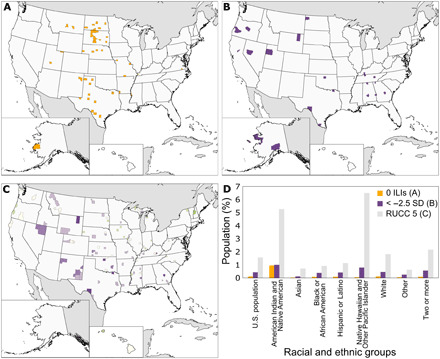
Counties that are the most underserved by ILIs. (**A**) Counties that do not have ILIs. (**B**) Counties with ILI residuals in the lowest 0.5% (σ < −2.5). (**C**) Non-metro, non-adjacent counties with urban populations over 20,000 (RUCC 5) with colors indicating standard deviations of log ILI residuals and the interaction of log population density and poverty percentage as in Fig. 2A. (**D**) Racial and ethnic percentages in underserved counties. U.S. population is the percentage of the general population in each underserved group of counties for comparison with the racial and ethnic groups. Bar plot values are in table S1.

We further examined the demographics of counties that we identified as underserved. There are 48 counties with no ILIs (Fig. 3A), 21 counties in the lowest 0.5% of ILI residuals (Fig. 3B), and 92 counties in RUCC 5 (Fig. 3C). Five RUCC 5 counties also have residuals in the lowest 0.5%. These counties are Campbell, WY; Garfield, OK; Lamar, TX; Val Verde, TX; and Laurens, GA. They include a total of 256,365 people, and each occurs in a different poverty category from 1 to 5, respectively. In all, these 156 underserved counties (5.0%) are home to 6,472,211 people (2.0% of the U.S. population).

Underserved counties occur across the Rural-Urban Continuum depending on the group. Counties without ILIs are mostly (48%) in RUCC 9—completely rural or urban with less than 2500 people and not adjacent to a metro area. Counties with ILI residuals in the lowest 0.5% are mostly the mid-metro counties with 250,000 to 1,000,000 residents (RUCC 2; 24%) or the largest non-metro counties with an urban area of 20,000 people that are not adjacent to a metro area (RUCC 5; 24%). Counties in RUCC 5 are micropolitan areas with urban cores of 10,000 to 50,000 people (*25*), such as Gillette, WY; Carlsbad, NM; and Elko, NV (Fig. 3C).

All underserved counties occur across the poverty categories. Most counties without ILIs (40%) are in poverty category 1 (0 to 10.1% poverty) with a mean poverty of 17%. Most counties with ILI residuals in the lowest 0.5% (29%) and counties in RUCC 5 (39%) are in poverty category 3 (14.2 to 18.4% poverty). Counties in these groups have a mean of 18% of people living in poverty.

The three underserved groups of counties include a small percentage of the overall U.S. population but larger percentages of Indigenous populations (Fig. 3D and table S1). Counties without ILIs include just 0.10% of the U.S. population but 0.95% of the American Indian or Alaskan Native populations (χ^2^ = 7.02, *P* < 0.01). Only 0.43% of the U.S. population lives in counties with ILI residuals in the lowest 0.5%, yet 1.0% of the American Indian or Alaskan Native population (χ^2^ = 0.762, *P* = 0.383) and 0.79% of the Native Hawaiian and Other Pacific Islander population (χ^2^ = 0.316, *P* = 0.574) reside in these counties. Similarly, although 1.6% of the U.S. population lives in RUCC5 counties, 5.3% of the American Indian or Alaskan Native population (χ^2^ = 8.72, *P* < 0.01) and 6.5% of the Native Hawaiian and Other Pacific Islander population (χ^2^ = 15.6, *P* < 0.01) live in RUCC5 counties.

## DISCUSSION

**Fig. 4:**
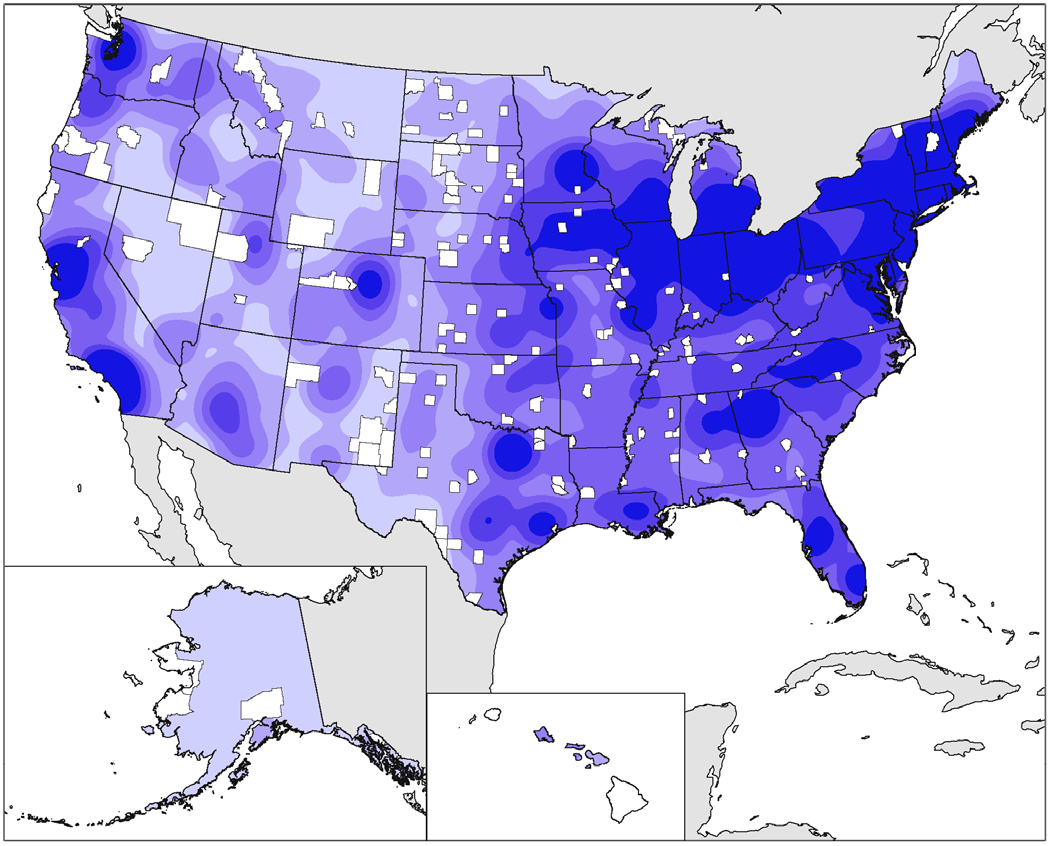
ILI density from Fig. 1A with underserved counties from Fig. 3 (A to C) highlighted in white.

Many of the counties underserved by ILIs are, as expected, in regions of low ILI density, and the largest counties by area also occur in areas of lowest ILI density (Fig. 4). This increases the challenge of providing informal STEM learning opportunities in these areas. Some of the underserved counties, particularly in the eastern part of the United States, are in regions with neighboring counties with high ILI density; residents of these underserved counties may take advantage of informal learning resources in nearby counties depending on their access to transportation. Conversely, ILI practitioners may be able to use these learning resources to target nearby counties with fewer ILIs and increase opportunities for participation closer to home for neighboring county residents.

Although establishment of new ILIs within the identified gaps would be ideal to increase learning opportunities, we appreciate that new infrastructure is likely to require time and substantive resources that may not be readily available. Partnerships between educational and community organizations can broaden participation of underserved populations (*26*), and ILIs are well situated to play a role in these partnerships because of their geographically widespread distributions. Many of the ILIs that occur within these underserved counties are libraries; in particular, libraries are 75% of the ILIs found in RUCC5 counties. Of the ILIs included in this study, libraries are a large portion (82%), and our results highlight the presence of libraries more so than the other ILI types. Their predominance and ability to provide access to in-person programming and digital resources position libraries as critical contributors to the ILI community and landscape (*27*, *28*). When other ILIs, such as museums and botanical gardens, are further away, libraries can facilitate STEM learning in these underserved communities (*27*–*29*).

Note that visitor experiences vary across ILI types and even across institutions within the same ILI type. For instance, libraries may not provide the same STEM learning opportunities as other institutions, such as museums and national parks. NPS lands and FSMLs play a unique role in the ILI landscape by using their resources to provide place-based informal STEM education to the public (*30*–*34*), and these institutions reach geographic areas with fewer ILIs of other types (Fig. 1B). FSMLs are also unique, because they can easily incorporate scientists in their outreach programming (*30*, *31*). Interacting with a scientist or STEM professional can result in positive learning outcomes for participants, such as increased interest in science, learning, and awareness of STEM careers (*35*, *36*). Even at National Parks, scientists are more able to interact with visitors at field stations located within the parks than at visitor centers (*37*). Unlike marine laboratories, primarily located along the coast, biological field stations occur across the country’s interior and can reach more geographically widespread and isolated populations (*31*).

Our analysis of ILIs in the United States did not include city, regional, or state parks, forests, and preserves because of inconsistent availability of data and disparate data formats across local, regional, and state governmental systems. The inclusion of these regional and local ILIs would increase the overall density of the ILI landscape as well as the density of parks. However, we do not expect that these additions would substantially change the underserved counties identified here. It will be interesting for local and regional studies to consider the differences in park systems across levels of government.

By identifying spatial inequalities in the informal STEM learning landscape, our analysis is an important step in addressing informal STEM learning inequity. Gaps in the ILI landscape represent opportunities for resource investment and capacity building in informal STEM education (*15*). Because those counties without ILIs, those with too few ILI opportunities, and those outside of metro areas have a distance-based obstacle to informal STEM learning, we recommend targeted efforts to reach these three groups of underserved counties (Fig. 3). Yet, presence of ILIs in a geographic location does not indicate that the location is equitably accessible to all potential visitors. Where there are ILIs, additional access barriers include financial costs, language and cultural difficulties, and degree of interest (*17*, *21*, *24*).

The counties identified as underserved have fewer than expected ILIs and also larger percentages of Indigenous groups (Fig. 3). Indigenous peoples are underrepresented in STEM careers (*38*) and are often disinterested in Western science because it conflicts with their cultural identity (*39*). Community-based and place-based learning are two methods that can promote Western science and Indigenous knowledge as distinct and complementary (*38*, *39*). Underserved counties would benefit from increased investment in informal STEM learning opportunities that meaningfully integrate Indigenous knowledge into science education programs (*40*). By providing engaging learning experiences for surrounding communities, ILIs can foster an increase in underrepresented groups in STEM careers and, more broadly, a more scientifically literate population that can rely on scientific findings to inform decision-making and influence policies in areas such as health, technology, and the environment (*1*, *4*, *5*).

## MATERIALS AND METHODS

### Experimental design

To map U.S. ILIs, we extracted locality data from the Institute of Museum and Library Services’ Museum Universe Data File (*41*). Records were cleaned to remove duplicates and were maintained for different institutions at the same geographic location, e.g., Southwest Minnesota State University (SMSU) Museum of Natural History and SMSU Planetarium. From this, geographic locations of 2962 STEM-related ILIs were compiled, including 1010 arboretums, botanical gardens, and nature centers (BOT); 1490 children’s museums, natural history museums, natural science museums, science and technology museums, and planetariums (MUS); and 462 zoos, aquariums, and wildlife conservation centers (ZOO). Geographic locations of additional ILIs in our analysis included 16,720 public central and branch libraries (LIB) extracted from the Public Library Survey’s Outlet Data File (*42*). Libraries were removed if they were listed as bookmobiles or books-by-mail only, reported as temporary or permanent closure, or located in outlying territories of the United States. Geographic locations of NPS lands included 167 national parks, national monuments, national preserves, and national seashores (*43*). Centroid points were used to represent NPS lands because informal learning activities are often available throughout the park. Last, geographic locations of 435 FSMLs were compiled from the Organization of Biological Field Stations (*44*) and National Association of Marine Laboratories (*45*). In all, geographic locations of 20,284 ILIs were included in this study and are available in data file S1. We recognize that our dataset does not include all ILIs available to the public in the United States. For instance, while we have included NPS lands, we do not have data on city, regional, or state parks. These locations often provide educational programming and increase the availability of informal learning opportunities in many areas.

We sourced population data at the county level from the American Community Survey (ACS) 2017 5-year estimate (*46*) and extracted population density, measures of poverty, and percentage of populations from each racial and ethnic group. Population density was calculated as the census number divided by the land area for each county (individuals per square kilometer) and was transformed to natural log for analysis. For each county, we extracted an RUCC from the U.S. Department of Agriculture Economic Research Service (*47*). Nine RUCC categories were based on county population density and metropolitan influence (*48*). The first three categories (RUCC 1 to 3) were metro counties, and the remaining six (RUCC 4 to 9) were non-metro counties that were either adjacent or not adjacent to a metro area (table S2) (*48*). Adjacency was defined as a shared border with a metro area and at least 2% of workers commuting into the central counties of the larger metro area (*48*). Poverty was determined at the family level by comparing income over the previous 12 months to set thresholds that vary depending on the size of the family (*49*). Percentage of poverty was binned into six categories from lowest (1) to highest (6) using Jenks natural breaks (table S3) (*50*). Racial and ethnic data from the ACS 2017 5-year estimate dataset (*46*) were used to calculate population percentages. These county-level data are available in data file S2.

### Statistical analysis

To determine the density of ILIs, we calculated a kernel density surface of ILIs per square kilometer with a cell size of 1000 within a World Geodetic System 1984 spatial reference system. At each ILI location, the ILI kernel density was extracted from the kernel density surface to calculate the mean density, SD, and SE for all ILIs and for each ILI type (BOT, FSML, LIB, MUS, NPS, and ZOO). The ILI densities for each type were compared using a one-way analysis of variance (ANOVA) test to determine statistical differences between the types. ILI type and kernel densities associated with each ILI are available in data file S1.

Next, we calculated a simple density surface of ILIs per county area (km^2^) that we could compare to county population density and percentage of poverty. We modeled the influence of log population density and poverty percentage on ILI density with a generalized linear model that included log population density, poverty percentage, and their interaction as factors, along with a rational quadratic correlation structure to account for spatial autocorrelation of the variables. McFadden’s pseudo-*R*^2^ was used to evaluate the models. This statistic indicates a good fit when 0.2 ≤ *R*^2^ (*51*). We compared the residuals of this model across RUCCs to determine the geographic element of equality of access relative to population density. We also compared the residuals across poverty categories. A positive residual value indicates more than expected opportunities based on the generalized linear model, and a negative residual value indicates fewer than expected opportunities based on the same generalized linear model. Residuals were evaluated across RUCC categories and poverty categories using analyses of variance.

Three strategies were then used to identify groups of counties most underserved in the ILI landscape: (i) counties with no ILIs, (ii) 0.5% of counties with the greatest negative residuals defined as more than 2.5 SDs below the mean (i.e., they had the fewest ILIs relative to their expected number), and (iii) counties in the RUCC or poverty category with the greatest mean negative residual. The greatest mean negative residual indicated the group with the fewest opportunities relative to the expected. We calculated the percentages of the U.S. population and racial and ethnic groups in each of the three groups of underserved counties [(group population of underserved counties/total group population of the United States) × 100]. All analyses were performed in ArcMap and R (*52*, *53*).

## Supplementary Material

abb3819_Data_file_S1.xlsx

abb3819_SM.pdf

abb3819_Data_file_S2.xlsx

## SUPPLEMENTARY MATERIALS

Supplementary material for this article is available at http://advances.sciencemag.org/cgi/content/full/6/41/eabb3819/DC1
