# Supplementary material for: Spatial inequalities leave micropolitan areas and Indigenous populations underserved by informal STEM learning institutions
Source: Sci Adv. 2020 Oct 9;6(41):eabb3819. doi: 10.1126/sciadv.abb3819 (PMC8262761; doi:10.1126/sciadv.abb3819)
Supplement: abb3819_SM.pdf [file abb3819_SM.pdf]

Science Advances
AAAS

advances.sciencemag.org/cgi/content/full/6/41/eabb3819/DC1

Supplementary Materials for

Spatial inequalities leave micropolitan areas and Indigenous populations underserved by informal STEM learning institutions

Rachel A. Short*, Rhonda Struminger, Jill Zarestky, James Pippin, Minna Wong, Lauren Vilen, A. Michelle Lawing

*Corresponding author. Email: rachel.a.short@tamu.edu

Published 9 October 2020, *Sci. Adv.* **6**, eabb3819 (2020)
DOI: 10.1126/sciadv.abb3819

The PDF file includes:

Tables S1 to S6
Legends for data files S1 and S2

Other Supplementary Material for this manuscript includes the following:

(available at advances.sciencemag.org/cgi/content/full/6/41/eabb3819/DC1)

Data files S1 and S2

Supplementary Materials

Table S1. Racial and ethnic groups in underserved counties for Figure 3D. Column names correspond to the three underserved groups of counties in Figure 3: (**A**), Counties that do not have ILIs, (**B**), Counties with ILI residuals in the lowest 0.5%, and (**C**), Non-metro, not adjacent counties with urban populations over 20,000 (RUCC 5). Population values are from the US Census Bureau (*46*).

Group	Population	Population in Figure 3A (%)	Population in Figure 3B (%)	Population in Figure 3C (%)
US population	321,004,407	0.10	0.43	1.57
American Indian and Alaska Native	2,098,763	0.95	1.00	5.26
Asian	16,989,540	0.03	0.11	0.72
Black or African American	39,445,495	0.08	0.39	0.92
Hispanic or Latino	56,510,571	0.09	0.42	1.14
Native Hawaiian and Other Pacific Islander	515,522	0.05	0.79	6.50
White	197,277,789	0.11	0.46	1.82
Two or more races	7,451,295	0.05	0.25	0.62
Other	715,432	0.09	0.56	2.17

Table S2. Rural-Urban Continuum Codes with population totals from the ACS 2017 5-year estimate dataset (*46, 49*).

RUCC	Metro designation	County population	Metro adjacent	Number of counties	Total population
1	Metro	Metro area of 1 million or more	--	432	177,601,619
2	Metro	Metro area of 250,000 to 1 million	--	379	68,242,203
3	Metro	Metro area of fewer than 250,000	--	356	29,067,253
4	Non-metro	Urban area of 20,000 or more	Yes	214	13,549,273
5	Non-metro	Urban area of 20,000 or more	No	92	5,028,805
6	Non-metro	Urban area of 2,500 to 19,999	Yes	593	14,642,448
7	Non-metro	Urban area of 2,500 to 19,999	No	433	8,176,439
8	Non-metro	Completely rural or urban area with less than 2,500	Yes	220	2,122,539
9	Non-metro	Completely rural or urban area with less than 2,500	No	424	2,573,828

Table S3. Poverty categories using by Jenks' Natural Breaks of percentage of poverty. Maximum value is 56.7%. Poverty data and population values are from the US Census Bureau (*46*).

Poverty category	Percentage of poverty	Total population
1	0 – 10.1	82,305,003
2	10.2 – 14.1	95,747,420
3	14.2 – 18.4	100,696,255
4	18.5 – 23.5	28,811,267
5	23.6 – 31.4	1,229,241
6	> 31.5	1,172,384

Table S4. Quantile breaks for maps in Figures 1A and 1C-1H. Break values are the maximum ILI value per 1000 km^2 of each quantile.

Quantile	All	NPS	FSML	ZOO	MUS	LIB	BOT
1	0.36	0.0044	0.0048	0.0061	0.012	0.10	0.013
2	0.73	0.0064	0.012	0.017	0.049	0.41	0.033
3	1.6	0.0094	0.021	0.029	0.089	1.0	0.062
4	2.5	0.014	0.033	0.045	0.14	1.9	0.11
5	4.0	0.022	0.061	0.064	0.24	3.2	0.19
6	31	0.75	0.14	0.26	1.0	26	0.84

Table S5. Mean, standard deviation, and standard error of ILI density for each type. Mean per 1000 km^2 is displayed in Figure 1B.

Type	Mean	Standard deviation	Standard error
ALL	6.5	6.5	0.046
NPS	2.7	5.5	0.43
FSML	4.5	4.6	0.22
ZOO	6.2	6.5	0.30
MUS	6.5	6.4	0.17
LIB	6.5	6.6	0.051
BOT	7.2	6.9	0.22

Table S6. Standard deviation values for residuals for Figure 2A. Residuals are from a generalized linear model between log ILI density and the interaction of log population density and poverty percentage. Values are the maximum residuals associated with each standard deviation bin.

Standard deviation	Residuals
< -2.5	< -1.59
-1.5	-0.922
-0.5	-0.259
0.5	0.404
1.5	1.07
> 1.5	> 2.20

Data file S1. Informal learning institution data including type, location, and density data. Data sources are provided in the Materials and Methods, and metadata are provided within the data file.

Data file S2. County-level data including population, poverty, and informal learning institution data. Data sources are provided in the Materials and Methods, and metadata are provided within the data file.
